# COVID-19 Detection via Ultra-Low-Dose X-ray Images Enabled by Deep Learning

**DOI:** 10.3390/bioengineering10111314

**Published:** 2023-11-14

**Authors:** Isah Salim Ahmad, Na Li, Tangsheng Wang, Xuan Liu, Jingjing Dai, Yinping Chan, Haoyang Liu, Junming Zhu, Weibin Kong, Zefeng Lu, Yaoqin Xie, Xiaokun Liang

**Affiliations:** 1 Shenzhen Institute of Advanced Technology, Chinese Academy of Sciences, Shenzhen 518055, China; isahsalim@siat.ac.cn (I.S.A.); ts.wang@siat.ac.cn (T.W.); xuan.liu@siat.ac.cn (X.L.); jj.dai@siat.ac.cn (J.D.); yp.chan@siat.ac.cn (Y.C.); yq.xie@siat.ac.cn (Y.X.); 2 Department of Biomedical Engineering, Guangdong Medical University, Dongguan 523808, China; na.li3@gdmu.edu.cn (N.L.); 16620777686@163.com (H.L.); 13266024986@163.com (J.Z.); 18219310888@163.com (W.K.); 18813713335@163.com (Z.L.)

**Keywords:** COVID-19, ultra-low-dose, chest X-ray images, deep learning

## Abstract

The detection of Coronavirus disease 2019 (COVID-19) is crucial for controlling the spread of the virus. Current research utilizes X-ray imaging and artificial intelligence for COVID-19 diagnosis. However, conventional X-ray scans expose patients to excessive radiation, rendering repeated examinations impractical. Ultra-low-dose X-ray imaging technology enables rapid and accurate COVID-19 detection with minimal additional radiation exposure. In this retrospective cohort study, ULTRA-X-COVID, a deep neural network specifically designed for automatic detection of COVID-19 infections using ultra-low-dose X-ray images, is presented. The study included a multinational and multicenter dataset consisting of 30,882 X-ray images obtained from approximately 16,600 patients across 51 countries. It is important to note that there was no overlap between the training and test sets. The data analysis was conducted from 1 April 2020 to 1 January 2022. To evaluate the effectiveness of the model, various metrics such as the area under the receiver operating characteristic curve, receiver operating characteristic, accuracy, specificity, and F1 score were utilized. In the test set, the model demonstrated an AUC of 0.968 (95% CI, 0.956–0.983), accuracy of 94.3%, specificity of 88.9%, and F1 score of 99.0%. Notably, the ULTRA-X-COVID model demonstrated a performance comparable to conventional X-ray doses, with a prediction time of only 0.1 s per image. These findings suggest that the ULTRA-X-COVID model can effectively identify COVID-19 cases using ultra-low-dose X-ray scans, providing a novel alternative for COVID-19 detection. Moreover, the model exhibits potential adaptability for diagnoses of various other diseases.

## 1. Introduction

The world is still facing an unprecedented public health crisis as the COVID-19 pandemic, caused by the severe acute respiratory syndrome coronavirus 2 (SARS-CoV-2), continues to ravage. This global health crisis has resulted in a staggering loss of human lives and has provoked profound socio-economic disruption with far-reaching consequences across all aspects of human existence [1,2]. As of 10 March 2023, the number of confirmed COVID-19 cases worldwide had reached an alarming figure of 676,609,955, with a devastating death toll of 6,881,955 (https://coronavirus.jhu.edu/ (accessed on 10 March 2023)). These distressing statistics underscore the acute severity of the pandemic, highlighting the immense burden it has placed on healthcare systems globally and the magnitude of the destruction it has caused.

The complexity of COVID-19 as a disease is evident in its wide range of clinical manifestations, which can include common symptoms such as cough, fever, fatigue, and shortness of breath, as well as the more distinctive symptom of anosmia or loss of taste or smell. In its severe form, the disease can lead to life-threatening complications such as multi-organ failure, septic shock, and pneumonia. The need for hospitalization is often prompted by these severe manifestations, which tragically result in a significant number of fatalities [3,4]. Given the multifaceted symptoms and the potential for rapid deterioration of patients’ health, the medical complexity of COVID-19 highlights the crucial importance of swift and accurate diagnosis as a key component of effective pandemic management.

To effectively address this global health crisis, the scientific and medical communities have developed a diverse array of testing methods for COVID-19 detection. These methods encompass various techniques, including serologic, nucleic acid, antigenic, and ancillary tests, each playing a distinct and crucial role in the overall healthcare response [5]. However, despite these advancements, the absence of a universally effective detection technique remains a significant barrier to halting disease transmission. The variability in sensitivity and specificity across different testing methods complicates their reliability and accuracy, presenting persistent obstacles in managing and containing the spread of the disease.

The real-time reverse transcription polymerase chain reaction (RT-PCR) has become the predominant diagnostic method for detecting COVID-19 among various healthcare response frameworks. It is designed to identify the presence of SARS-CoV-2 RNA in respiratory samples, typically collected via nasopharyngeal swabs or sputum samples. RT-PCR is widely recognized as the most reliable and accurate testing approach for COVID-19, often referred to as the “gold standard” [6]. However, despite its prominence, the RT-PCR testing process is hindered by several intrinsic limitations that undermine its overall effectiveness. Conducting an RT-PCR test is notably labor intensive, requiring skilled personnel and strict adherence to complex protocols. The time-consuming nature of the procedure often leads to diagnostic delays, potentially resulting in delayed initiation of necessary treatment. Moreover, the process raises environmental concerns due to the substantial volume of medical waste it generates, raising questions about its sustainability. Additionally, the high costs associated with RT-PCR testing, including expenses for test kits and necessary equipment, limit its widespread application, particularly in economically disadvantaged regions [7]. Compounding these challenges, the sensitivity of RT-PCR tests can vary depending on the methods of sample collection and may decrease over time due to the rapid mutations and genetic heterogeneity of SARS-CoV-2. Operational complexities and logistical hurdles can impede the broad-scale deployment of RT-PCR tests, especially in densely populated areas, intensifying the global challenges in managing the pandemic.

In the quest for alternative testing methods to RT-PCR, chest X-ray (CXR) imaging has demonstrated significant potential in facilitating the detection of COVID-19 [8,9]. CXR images can reveal specific alterations in lung tissue associated with the disease, such as the appearance of ground-glass opacities [10]. These opacities, manifested as hazy or fuzzy areas often localized in the lower regions of the lungs, provide critical diagnostic indicators for COVID-19, emphasizing the essential role of CXR images in the diagnostic process [11]. Compared to other diagnostic tools like RT-PCR, CXR images offer numerous advantages, including cost-effectiveness, immediate availability, reduced risk of cross-infection, minimized radiation exposure, and widespread accessibility. These attributes significantly contribute to accelerating and optimizing the COVID-19 diagnostic process, playing a critical role in preventing further disease dissemination. However, conventional X-rays do expose patients to some level of radiation, necessitating the use of ultra-low-dose X-ray images for COVID-19 detection.

Minimizing additional radiation exposure is a critical consideration for patient safety. In this context, ultra-low-dose X-ray images emerge as a promising direction for quick and recurrent detections, potentially introducing a significant paradigm shift in the management of viral transmission routes. Despite the considerable advancements in the detection methodologies conceptualized and developed in response to the COVID-19 pandemic, the absence of a universally effective detection strategy continues to present a significant roadblock in mitigating disease transmission. Therefore, a comprehensive understanding of the limitations of these methodologies is crucial for improving their effectiveness and bolstering collective efforts to combat the COVID-19 pandemic. COVID-19 is a global pandemic, and rapid and accurate detection of the virus is crucial for controlling its spread. This study addresses the urgency of detection, which is vital for identifying and isolating infected individuals promptly.

In the context of COVID-19 detection, researchers have exhibited a pronounced interest in leveraging pre-trained DL models due to their inherent capability to extract salient features and discern intricate patterns within radiological images. Prominent architectures in this domain, including AlexNet [12], Xception [13], ResNet [14], DenseNet [15], VGG [16], MobileNet [17], and Inception [18], have been subject to scrutiny for their architectural attributes such as depth, robustness, and input size. The selection of an appropriate architecture hinges on a meticulous examination of these properties.

However, the deployment of DL models necessitates substantial volumes of data, which is a requirement that presents challenges in the context of COVID-19 research. The novelty of the virus has resulted in a dearth of standardized data, confounding diagnostic efforts. Furthermore, image datasets sourced from COVID-19 patients often exhibit issues such as mislabeling, noise contamination, incompleteness, and overall clarity deficits. The presence of extensive and heterogeneous datasets poses formidable challenges during model training, encompassing problems related to data redundancy, missing values, and data sparsity.

In order to tackle these difficulties, we present ULTRA-X-COVID, an advanced deep learning (DL) model specifically developed for the identification of COVID-19 using ultra-low-dose X-ray images. Our innovative approach involves a DL framework that effectively reduces radiation exposure while maintaining functionality. The study encompasses a substantial and diverse dataset, including 30,882 X-ray images obtained from approximately 16,600 patients across 51 countries. The absence of overlap between the training and test sets enhances the robustness of the research. This research is vital as it addresses the urgent need for efficient, safe, and accurate COVID-19 detection methods. It leverages cutting-edge deep learning technology and prioritizes patient well-being, making it a significant contribution to public health. The primary achievements of our study are outlined as follows:

- We propose ULTRA-X-COVID-Net, an innovative model that dramatically reduces radiation exposure. This model demonstrates remarkable performance in detecting COVID-19, thereby enhancing the efficiency and effectiveness of disease management.
- We are the first to develop a deep U-Net model specifically designed for denoising CXR images. We have significantly improved the standard U-Net architecture’s skip-connection method to enhance denoising performance.
- We have provided comprehensive experimental results that validate the effectiveness of the proposed method. Compared to the state-of-the-art methods, our approach demonstrates excellent performance in detecting COVID-19 from X-ray images.
- The ULTRA-X-COVID model’s rapid prediction time of only 0.1 s per image is a significant novelty as it ensures quick diagnosis without compromising accuracy.

The organization of the rest of the paper is as follows: Section 2 provides materials and methods on COVID-19 detection. The result are presented in Section 3. A discussion about the results is available in Section 4. Finally, Section 5 concludes the paper.

## 2. Materials and Methods

### 2.1. Dataset Collection and Annotation

This multinational, multicenter retrospective cohort study utilized a vast dataset consisting of 30,882 X-ray images collected from approximately 16,600 patients across 51 countries. Out of this dataset, 16,690 images were obtained from patients with confirmed cases of COVID-19, while 14,192 images were sourced from patients who tested negative for the virus. The images have a resolution of 1024 × 1024 pixels.

To ensure unbiased evaluation, the dataset was divided into a testing set comprising 200 COVID-19-positive images from 178 patients and an equal number of COVID-19-negative images from 100 patients. The remaining images were reserved for training the model. It is worth emphasizing the importance of a balanced test set in providing an objective assessment of the model’s performance.

For the selection of test images, a random sampling approach was adopted from an international patient cohort assembled by the Radiological Society of North America [19]. These test images were meticulously annotated by an international consortium of scientists and radiologists to ensure accurate labeling. Care was taken to avoid any overlap between the training and test sets, thereby maintaining the integrity of the training and testing processes.

This dataset, notable for its extensive scale, currently stands as the largest publicly available benchmark dataset for confirmed COVID-19 cases in the literature [20]. Table 1 shows hyperparameter values of the dataset.

### 2.2. Data Augmentation

A data augmentation technique is used in the ULTRA-X-COVID model for COVID-19 detection to increase the diversity of the training dataset. It involves applying various transformations to the existing X-ray images, creating new training examples. These transformations can include rotations, flips, zooms, and adjustments in brightness and contrast. We used rotation within the range [−15, 15], translation in x- and y-axis within the range [−15, 15], horizontal flipping, scaling, and shear within the range 85–115%.

Data augmentation helps improve the model’s robustness and generalization, allowing it to perform better on unseen data, ultimately enhancing the accuracy and reliability of COVID-19 detection.

### 2.3. Description of the Proposed Model

In the context of U-Net and ResNet101, artificial neural networks (ANNs) are the foundational framework that underpins both architectures. A U-Net is a convolutional neural network architecture widely used in biomedical image segmentation tasks. It is characterized by a U-shaped architecture, hence the name “U-Net”. The U-Net architecture consists of a contracting path to capture context and a symmetric expanding path to achieve precise localization of objects in an image [21].

One of the key features of the U-Net architecture is its skip-connection method, which incorporates features from the contracting path and merges them with the corresponding layers in the expanding path. These skip-connections facilitate the flow of fine-grained information from early layers to later layers, helping the network preserve spatial details during the upsampling process [22].

On the other hand, ResNet101 demonstrates the power of deep ANNs in handling very deep networks with the aid of residual connections. Both U-Net and ResNet101 leverage ANNs to solve specific challenges in image analysis and computer vision [23].

**Figure 1:**
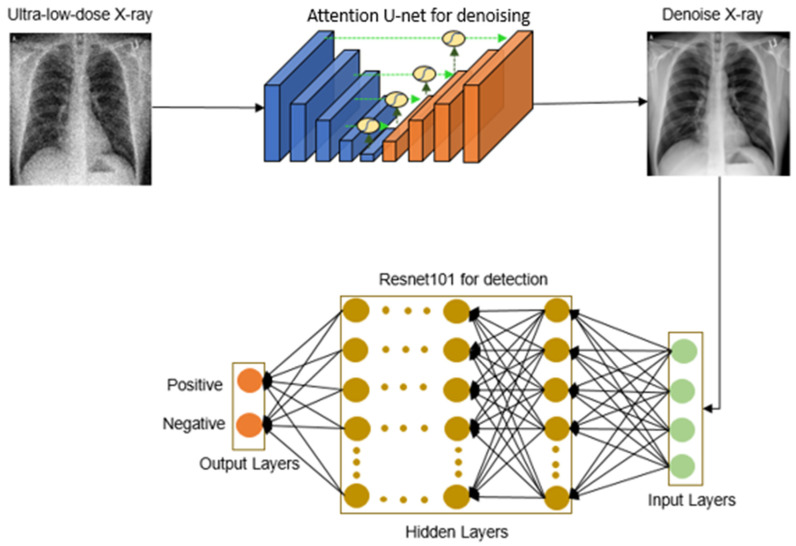
Schematic diagram of the proposed ULTRA-X-COVID model.

We introduce “ULTRA-X-COVID”, a model specifically designed for the detection of COVID-19 in ultra-low-dose X-ray images, as depicted in Figure 1. This system analyzes ultra-low-dose X-ray images of patients’ lungs by utilizing DL algorithms, providing a possibly novel strategy in the fight against the COVID-19 pandemic.

The operational workflow of the model involves processing ultra-low-dose X-ray images through an attention-based U-Net, generating denoised CXR images. These denoised images then undergo binary classification employing a deep residual convolutional neural network. With thousands of convolutional layers, this deep residual network effectively mitigates error rates and the vanishing gradient problem, ensuring robust and efficient performance.

The integration of DL principles within the model facilitates dependable detection, which is a critical feature in disease management. It highlights the potential of this powerful tool in expediting the identification of COVID-19 cases and, consequently, saving lives.

### 2.4. Denoising Network for Ultra-Low-Dose X-ray Imaging

The simulated low-dose image generation process involves transforming high-dose X-ray images into realistic low-dose counterparts while considering factors such as noise and radiation exposure levels. These simulated images play a crucial role in training and evaluating DL models for COVID-19 detection, enabling the development of safer and more effective diagnostic tools.

**Figure 2:**
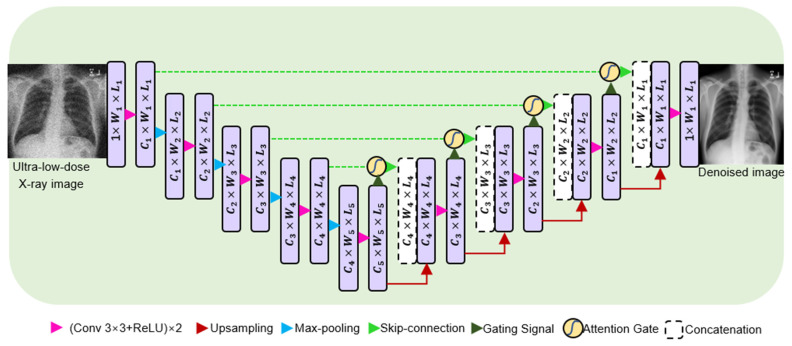
Block diagram of the proposed denoising network for ultra-low-dose X-ray images.

Our purpose-built denoising network for ultra-low-dose CXR images is depicted in Figure 2. We implemented the technique outlined in [24] to generate realistic ultra-low-dose X-ray images from their full-dose counterparts.

Within the encoding module, the number of image channels either decreases or increases by a factor of 2 at each successive level, as represented by (W3×L3=(W122)×(L122),C3=22×C1, F3=22×F1 ).

Contrastingly, the decoding module operates in reverse, altering the number of image channels in the opposite direction, either increasing or decreasing by a factor of 2 at each level. In these equations, parameters *W*, *L*, and *C* refer to the image height, width, and length, respectively, acquired from the attention U-Net. $F_{3}$ and $F_{1}$ represent the number of feature maps (filters) in the third and first layer of the U-Net, respectively. Similar to the previous equation, this equation states that $F_{3}$ is equal to 4 times $F_{1}$, which means that $F_{3}$ is four times the value of $F_{1}$.

Contrastingly, the decoding module operates in reverse, altering the number of image channels in the opposite direction, either increasing or decreasing by a factor of 2 at each level. In these equations, parameters *W*, *L*, and *C* refer to the image height, width, and length, respectively, which are acquired from the attention U-Net.

To generate ultra-low-dose X-ray images, we employed the methodology described in [24]. The simulated noise encompasses both signal-independent and signal-dependent components. The Additive White Gaussian Noise (AWGN) imitates the signal-independent electronic noise within the image domain, while the filtered AWGN introduced in the X-ray projection domain mimics signal-dependent quantum noise [25]. AWGN represents signal-independent electronic noise added to the image, and filtered AWGN in the X-ray projection domain simulates signal-dependent quantum noise that varies with X-ray intensity.

The process described pertains to simulating ultra-low-dose X-ray images rather than the creation of true physical images. We utilize a modeling approach that incorporates detector blur within the filtered Additive White Gaussian Noise (AWGN) to simulate the effects of quantum noise at ultra-low radiation doses. The process of creating ultra-low-dose X-ray images involves incorporating detector blur within the filtered AWGN to model quantum noise [26]. This process can be represented by the following equation:

(1) $$\mu_{l}=GAT^{-1}\left(GAT\left(\mu_{f}+k_{q}\times \eta_{q}\right)\right)+\eta_{e}$$

In this equation, $\mu_{f}$, and $\mu_{l}$ denote the full-dose X-ray image and the simulated low-dose X-ray image, respectively. The variables $\eta_{q}$ and $\eta_{e}$ represent AWGN applied in the GAT domain and image domain, respectively. GAT(·) refers to the generalized Anscombe transform, defined as:

(2) $$GAT\left(x\right)=\frac{2}{\alpha }\sqrt{\alpha \cdot x+\frac{3}{8}\alpha^{2}+\sigma_{n}^{2}}$$

Variables α and $\sigma_{n}$ are related to the gain mode of the detector and the standard deviation of electronic noise, respectively. These values can be ascertained from calibration measurements or system specifications. $GAT^{-1}\left(\cdot \right)$ represents the inverse GAT, and the filtering kernel $k_{q}$ is a Gaussian kernel of size 2 *×* 2.

### 2.5. Design and Implementation of the COVID-19 Detection Network

**Figure 3:**
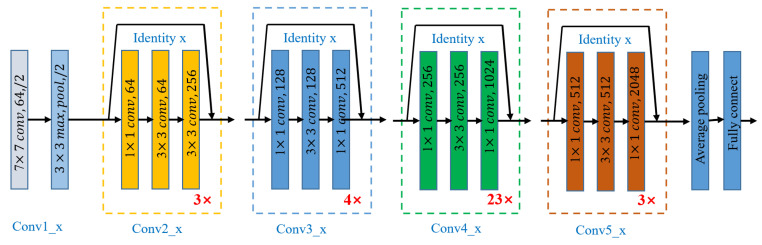
Architecture of the proposed detection network.

Our study introduces ULTRA-X-COVID, an advanced deep residual convolutional neural network model for COVID-19 detection. This model showcases exceptional capabilities in acquiring intricate image features. The architecture can be broken down as follows and as shown in Figure 3. Conv1_x: The image undergoes a 7 × 7 convolution (64 filters), capturing basic features. This is followed by a 3 × 3 max-pooling layer, reducing spatial dimensions. Conv2_x: Features a bottleneck structure repeated 3 times: a 1 × 1 (64 filters), 3 × 3 (64 filters), and another 1 × 1 convolution (256 filters). “Identity” connections are incorporated. Conv3_x: contains a pattern of 1 × 1 (128 filters), 3 × 3 (128 filters), and 1 × 1 (512 filters) convolutions, repeated 4 times with identity connections. Conv4_x: uses a bottleneck structure of 1 × 1 (256 filters), 3 × 3 (256 filters), and 1 × 1 (1024 filters), replicated 23 times with identity connections. Conv5_x: Features three bottleneck structures: 1 × 1 (512 filters), 3 × 3 (512 filters), and 1 × 1 (2048 filters). Identity connections are present. Average Pooling: a global average pooling layer reduces feature map dimensions. Fully Connected Layer: the final layer gives output predictions for COVID-19 detection.

During the training phase, the ULTRA-X-COVID model learns to distinguish patterns associated with COVID-19. We employed the pre-trained ResNet-101 model, loaded using the resnet101 function from MATLAB R2022a’s Neural Network Toolbox. The model accepts 2D images with dimensions 224 × 224 × 3 as input and employs the Adam optimizer algorithm. The hyperparameters were set for a mini batch size of 256, maximum iterations, and an initial learning rate. The model performs image detection with a stride of 2 using a fully connected layer and a softmax function. The detailed architecture of the ULTRA-X-COVID model is illustrated in Figure 3.

### 2.6. Implementation Details and Evaluation Metrics

The simulations were conducted on a Windows 10 operating system, utilizing two NVIDIA GeForce GTX XP graphics processing units, an Intel CPU E5-2697 v3, and 128 GB of RAM. The implementation was performed using PyTorch 1.7, which is an open-source framework for machine learning, and the Python programming language. Weight updates were executed over 100 epochs using the Adam optimizer with a learning rate of 10^−4^.

To assess the effectiveness of the ULTRA-X-COVID Net, various metrics were employed to evaluate the binary detection outcomes and the model’s efficiency. These metrics include

(3) $$\mathrm{True}\;\mathrm{positive}\;\mathrm{rate},\;\mathrm{recall}=\frac{\mathrm{TP}}{\mathrm{TP}+\mathrm{FN}}\times 100$$

(4) $$\mathrm{False}\;\mathrm{positive}\;\mathrm{rate}=\frac{\mathrm{FP}}{\mathrm{FP}+\mathrm{TN}}\times 100$$

(5) $$\mathrm{Precision}=\frac{\mathrm{TP}}{\mathrm{TP}+\mathrm{FP}}\times 100$$

(6) $$\mathrm{Accuracy}=\frac{\mathrm{TP}+\mathrm{TN}}{\mathrm{TP}+\mathrm{TN}+\mathrm{FP}+\mathrm{FN}}\times 100$$

(7) $$F1-\mathrm{Score}=\frac{2\times \mathrm{Precision}\;\times \mathrm{Recall}}{\mathrm{Precision}+\mathrm{Recall}}\times 100$$

(8) $$\mathrm{MCC}=\frac{\mathrm{TN}\times \mathrm{TP}-\mathrm{FN}-\mathrm{FP}}{\sqrt{\left(\mathrm{TP}+\mathrm{FP}\right)\left(\mathrm{TP}+\mathrm{FN}\right)\left(\mathrm{TN}+\mathrm{FP}\right)\left(\mathrm{TN}+\mathrm{FN}\right)}}\times 100$$

with True Positive (TP), False Positive (FP), True Negative (TN), False Negative (FN), Accuracy (ACC), Matthews Correlation Coefficient (MCC), and so on.

### 2.7. Statistical Analysis

The primary evaluation metric for assessing the discriminative ability of the prediction models was the area under the receiver operating characteristic curve (AUC). The statistical significance of differences between AUCs was determined using the DeLong test. To determine if the observed differences in sensitivities and specificities were statistically significant, McNemar’s test was utilized. A *p*-value less than 0.05 was considered statistically significant. The statistical analyses were conducted using the SciPy1.6.0, library in Python.

### 2.8. Computational Complexity Analysis

Our analysis focused on the U-Net-based denoising network and the ResNet-101-based COVID-19 detection network. We quantified their computational complexity in terms of parameters and FLOPs. The computational complexity stems from its depth and the varying number of channels in each layer. A ballpark figure situates its complexity in the order of billions of FLOPs for standard input sizes like 224 × 224. However, both architectures benefit significantly from parallel acceleration. Their inherent properties, like layer-wise parallelism and data parallelism, coupled with optimization techniques for matrix multiplication on GPUs, ensured efficient computation, meaning the ULTRA-X-COVID model strikes a balance between noise reduction, feature extraction, and prediction, while maintaining moderate computational complexity.

## 3. Results

### 3.1. Performance Analysis of the ULTRA-X-COVID Net Model

This study aims to comprehensively evaluate the effectiveness of different methodologies applied to full-dose and ultra-low-dose CXR images for detecting COVID-19. A comparative analysis is presented in Table 2, which includes evaluation metrics obtained from the established ResNet model and our novel approach, the ULTRA-X-COVID Net model. The datasets were used for each evaluation at a ratio of 80% training and 20% testing for full dose, low dose and ultra-low dose, respectively.

Up arrow (↑) usually represents an increase or improvement in the value of a metric for instance F1-Score and accuracy has increased compared to a previous measurement, while down arrow (↓) Conversely, used to signify a decrease or reduction in the value of a metric. If, for instance, FP and FN have decreased compared to a previous measurement, a down arrow is used to show the reduction.

Upon systematic analysis, the ULTRA-X-COVID Net model demonstrates exceptional performance when applied to ultra-low-dose CXR images. Notably, its predictions closely match those obtained from full-dose CXR images, showcasing its precision. In terms of evaluation metrics, the ULTRA-X-COVID Net model outperforms the full-dose ResNet model in several crucial areas, including False Positives (FP), True Negatives (TN), Precision, and Specificity. The corresponding metric values are as follows: FP = 2, TN = 198, Precision = 0.989, and Specificity = 0.990.

In contrast, the conventional ResNet model shows lackluster performance when applied to ultra-low-dose X-ray images. With an overall accuracy of 0.878, a reduced recall of 0.795, and an F1-Score of 0.866, the ResNet model falls short. The proposed ULTRA-X-COVID Net method demonstrates a significant capacity to generate predictions that closely align with those derived from full-dose CXR images. This ability remains consistent regardless of the dose of the CXR images, making it stand out. In certain evaluation metrics, our model even outperforms the results obtained from full-dose images. This outcome highlights the potential of ULTRA-X-COVID Net as a suitable alternative for COVID-19 detection using ultra-low-dose X-ray images, offering the critical advantage of reducing radiation exposure and mitigating the associated risks for patients.

**Figure 4:**
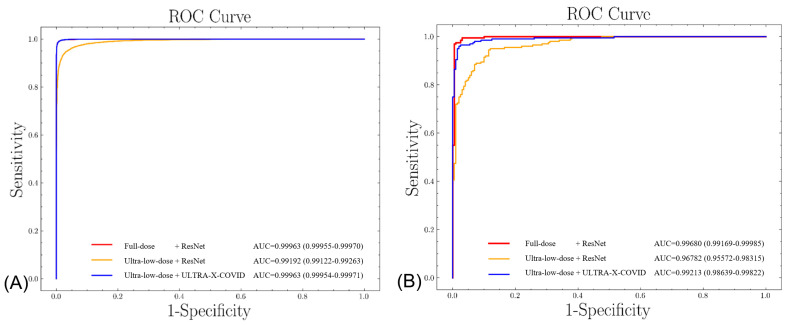
Receiver Operating Characteristics (ROC) curves using full-dose CXR images with different methods. (**A**) training set; (**B**) testing set.

Figure 4 presents the outcomes of our comparative analysis in a visual format, featuring two graphs representing the Area Under the Curve (AUC) performance of the training and test sets. Each graph provides a visual comparison of the performance of three different methods: Full dose + ResNet, Ultra-low dose + ResNet, and Ultra-low dose + Ultra-X-COVID Net. The red line corresponds to the Full dose + ResNet method, achieving AUC values of 0.99963 (0.99955–0.99970) on the training set and 0.99680 (0.99169–0.99985) on the test set. This result emphasizes the superior predictive efficacy of the Full dose + ResNet method when applied to high-dose X-ray images.

On the other hand, the yellow line represents the Ultra-low dose + ResNet method, with AUC values of 0.99192 (0.99122–0.99263) on the training set and 0.96782 (0.95572–0.98315) on the test set. These findings highlight the limitations in the predictive capability of the conventional ResNet method when utilized with ultra-low-dose X-ray images.

Lastly, the blue line indicates the performance of the Ultra-low dose + Ultra-X-COVID Net method, exhibiting AUC values of 0.99963 (0.99954–0.99971) on the training set and 0.99213 (0.98639–0.99822) on the test set. These values suggest that the predictive proficiency of the Ultra-X-COVID Net method on ultra-low-dose X-ray images is comparable to the performance of the Full dose + ResNet method on high-dose X-ray images.

**Figure 5:**
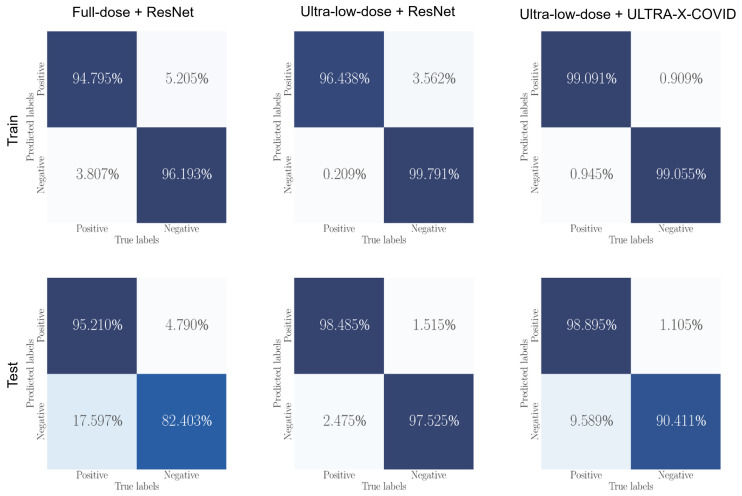
Normalized confusion matrix using full-dose and ultra-low-dose CXR images with different methods on the training and testing dataset.

The proposed Ultra-X-COVID Net method significantly outperforms the traditional ResNet method when applied to ultra-low-dose X-ray images, while nearly matching the predictive results of high-dose X-ray images. This outcome indicates the exceptional predictive performance of the Ultra-X-COVID Net method. Furthermore, this method exhibits great potential for reducing X-ray dosage, offering significant promise for practical applications. Additional supporting evidence for this conclusion is provided by the Normalized Confusion Matrix depicted in Figure 5.

### 3.2. Comparative Analysis of ULTRA-X-COVID-Net with Other Techniques

This subsection provides an in-depth performance evaluation of the proposed ULTRA-X-COVID Net model in comparison to recently developed deep learning-based methodologies. Table 3 presents a comparison demonstrating that the ULTRA-X-COVID-Net model outperforms contemporary methodologies across all evaluated metrics.

In assessing our model against current cutting-edge algorithms, we scrutinized several critical aspects, including model architecture, efficiency, invasiveness, scalability, and detection capabilities. The architecture of the ULTRA-X-COVID-Net model was meticulously designed to be highly efficient and minimally invasive. This unique construction enables the model to process extensive datasets in a time-efficient manner. Furthermore, our deep learning model has been specifically optimized for efficient detection, ensuring high-speed performance with minimal latency.

Therefore, in Table 3, we acknowledge the excellent work of S. Nafisah et al. [37] and Mukherjee et al. [30], which have achieved higher accuracy compared to ULTRA-X-COVID-Net model. Our model’s efficiency is exemplified by its ability to deliver rapid predictions even when dealing with ultra-low-dose images. By minimizing computational time and resource requirements, it can serve as a valuable tool for healthcare professionals. The model achieves an accuracy rate of 98%, an F1 score of 98%, a specificity of 98.5%, and an MCC of 96%. These results underline the potential of deep learning-based methodologies to make a significant contribution to the fight against COVID- 19. Such methodologies can provide accurate and efficient detection of the virus, serving as critical tools in ongoing and future pandemic mitigation strategies.

## 4. Discussion

This study presents an important contribution to the medical field by introducing a method for detecting COVID-19 using ultra-low-dose X-ray images, powered by DL. The accurate detection of COVID-19 is of utmost importance in the current pandemic, with CXR images serving as a crucial foundation for clinical scenarios and treatment strategies aimed at curbing the widespread transmission of the virus.

A notable aspect of our research is the systematic integration of DL to enhance the accuracy of COVID-19 detection in CXR images. We initially utilized a U-Net model to denoise the CXR images, effectively improving their quality by reducing extraneous noise. These denoised images were then fed into the ResNet-101 model, which is renowned for its layered structure and high efficacy, for the detection phase.

The practical value of the ResNet-101 model, demonstrated in the literature, lies in its ability to efficiently handle multiple layers. This capability enables it to analyze complex image structures, which proved crucial in our study for identifying COVID-19 signatures within CXR images.

To evaluate our proposed model, we employed the COVIDx dataset, which consists of 13,975 CXR images, including 13,870 images associated with COVID-19 and 105 images representing normal cases. The dataset was divided into an 80% subset for training and a 20% subset for testing. A key aspect of our experimental procedure was the careful selection of optimal statistical hyperparameters for the DL model. After extensive trials, we fixed the number of epochs at 100 and the mini batch size at 16, which were used throughout the model’s training process. Additionally, we fine-tuned the initial learning rate and weight parameters using the Adam optimization method.

Our ResNet-101 model served as the foundation for our binary detection system, and we assessed its effectiveness using a range of metrics, including accuracy, precision, recall, F1 score, and specificity. These metrics provide a comprehensive evaluation of the model’s detection capabilities, shedding light on its strengths and areas for potential improvement.

In Table 2, we present the performance outcomes of our full dose, low dose, and proposed models, as measured using various performance metrics. Notably, our proposed model outperformed other models, demonstrating exceptional accuracy that surpassed competing approaches. In comparison to state-of-the-art techniques, such as those by Dansana et al., 2020 [38], Jiang et al., 2021 [39], and Alam et al., 2021. [40], our proposed method achieved a significant enhancement in the accuracy of CXR image detection, achieving rates of 98.0%, 87.8%, and 94.3% for the full dose, low dose, and proposed models, respectively.

To gain further insights into the decision-making process of the DL model, we employed the Gradient-weighted Class Activation Mapping (Grad-CAM) technique. This technique allows us to generate heatmap visualizations that highlight the crucial regions within a CXR image that contribute to the final detection decision made by the ResNet-101 model. By doing so, we aimed to understand the model’s internal reasoning, identify key areas of the image that influence the detection outcome, and provide more interpretable insights into the model’s functioning. This transparency fosters trust and acceptance from users who rely on the model for COVID-19 detection.

**Figure 6:**
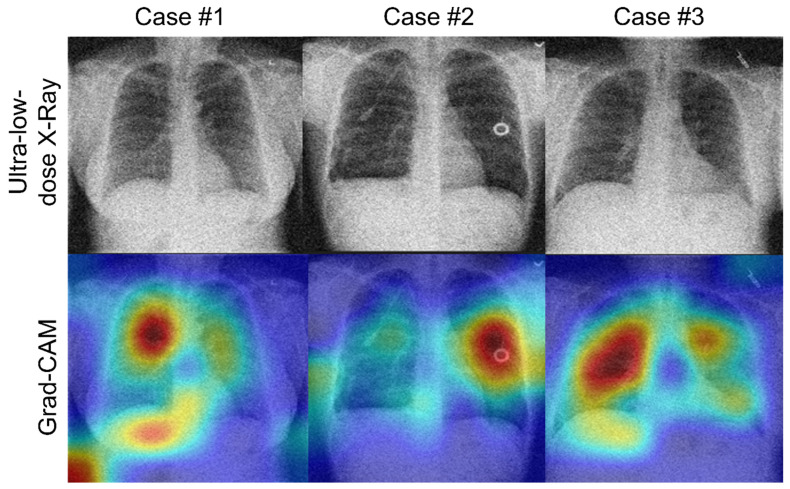
Sample images of Grad-CAM visualizations analysis. The colors represent the degree of activation, highlighting regions with better localization ability.

Figure 6 illustrates examples of the Grad-CAM visualizations, displaying noisy and denoised CXR images. It can be observed that the ResNet-101 model generates distinct, more compact features from denoised CXR images, while producing more diverse, dispersed features from the noisy CXR images. Interestingly, the denoised CXR images primarily focus on the lung region, whereas the noisy CXR images draw attention to irrelevant regions beyond the lung. This distinction emphasizes the robust lesion localization capability of our model, underscoring the importance of the denoising step for accurate detection.

### Limitations

Despite the promising potential of COVID-19 detection through ultra-low-dose CXR images facilitated by DL, several limitations and challenges need to be acknowledged.

Firstly, a crucial limitation lies in the strong dependence of the detection model on the quality and resolution of the denoised CXR images. It is essential to recognize that the effectiveness of the detection model is significantly influenced by the quality of these input images. Inferior quality or lower resolution images, resulting from inadequate scanning equipment or poor image handling and storage, can significantly degrade the model’s performance. While these challenges exist, we have implemented measures to mitigate them and are actively refining our model with ongoing data collection.

In summary, the quality of ultra-low dose X-ray images significantly impacts the accuracy of COVID-19 detection. Therefore, maintaining device readiness, optimizing image quality, and reducing noise are essential for reliable diagnoses and the early detection of COVID-19-related lung abnormalities.

Secondly, the availability and distribution of CXR machines pose another significant obstacle. The uneven global distribution of such technology, particularly in remote or under-resourced areas, limits the widespread implementation of this approach. It is important to note that without broad availability and proper functioning of these machines, the effectiveness of the proposed detection model will be significantly hindered.

The third limitation pertains to the potential inefficiency of the model in identifying COVID-19 in individuals who exhibit asymptomatic or mild symptoms. Since the manifestation of the disease in CXR images is closely related to the severity of symptoms, the model may lack sensitivity in detecting these asymptomatic or mildly symptomatic cases. Consequently, there is a risk of false negatives, potentially leading to the spread of the virus by individuals who are unaware of their condition.

Lastly, the model’s proficiency in accurately distinguishing COVID-19 in patients with underlying lung diseases poses a considerable challenge. These pre-existing lung conditions may display radiographic patterns on the CXR images that bear a striking resemblance to those generated by COVID-19, creating ambiguity in the interpretation. This can potentially lead to false positives, resulting in inaccurate diagnoses and inappropriate or delayed treatment.

## 5. Conclusions

The integration of ultra-low-dose X-ray images with DL techniques for COVID-19 detection holds tremendous promise in the fight against the ongoing pandemic. This approach offers several notable advantages, including reduced radiation exposure for patients and rapid diagnosis and prediction time in identifying COVID-19 cases.

This paper introduces the ULTRA-X-COVID model, which is a method for detecting COVID-19 using ultra-low-dose X-ray images and DL techniques with the COVIDx dataset. It highlights the advantages of reduced radiation exposure, efficiency, and speed. The performance of the model was evaluated in terms of precision, sensitivity, specificity, F1 score, MCC, and ROC. The ULTRA-X-COVID model achieved an accuracy of 94.3%, a specificity of 99%, an F1 score of 88.9%, and a precision of 98.9% for binary detection. The reduction in radiation exposure remains a prospective advantage of our approach. However, not only does our proposed model surpass some existing methods in terms of accuracy, but it also exhibits capability, which is crucial for radiologists and other medical professionals to gain a better understanding of COVID-19-related aspects. This research could play a significant role in effectively managing the COVID-19 pandemic and improving overall public health. Furthermore, the approach can be efficiently adapted to diagnostics of various other diseases.

## 6. Patents

The work reported in this manuscript has resulted in a patent.

## Figures and Tables

**Table 1:** Summarizing key hyperparameter values of the experiment.

Characteristics	Original Dataset	Transformed Dataset
Dataset size	30,882 X-ray images	30,882 X-ray images (after data augmentation)
Data types	X-ray images, labeled COVID/non-COVID	Augmented X-ray images, labeled COVID/non-COVID
Image Resolution	1024 × 1024	1024 × 1024
Data Split	Training: 80%, Testing: 20%	Training: 80%, Testing: 20%
Data augmentation	None	Random rotations, flips, brightness adjustments.
Full dose	10,000 images	10,000 images
Low dose	8000 images	8000 images
Ultra-Low dose	5882 images	5882 images

**Table 2:** Test results of three classifiers trained on full dose and our denoised X-ray images.

	Method	TP↑	FP↓	TN↑	FN↓	Accuracy↑	Precision↑	Recall↑	F1-Score↑	Specificity↑	MCC ↑
**Full dose**	ResNet	195	3	197	5	0.980	0.985	0.975	0.980	0.985	0.960
**Low dose**	ResNet	159	8	192	41	0.878	0.952	0.795	0.866	0.960	0.765
**Ultra-Low Dose**	ULTRA-X-COVID Net	179	2	198	21	0.943	0.989	0.895	0.940	0.990	0.889

**Table 3:** Comparison of the proposed ULTRA-X-COVID Net with other techniques.

Previous Study	Model	F1-Score%	Accuracy%	Specificity%	MCC
Sethy and Behra et al. [26]	ResNet50, SVM	95.52	95.38	93.47	90.76
Minaee et al. [27]	ResNet, DenseNet	-	-	90.0	-
Narin et al. [28]	ResNet, Inception	-	96.1	-	-
Hemdan et al. [29]	AlexNet	89	90	-	-
Mukherjee et al. [30]	Shallow CNN	99.69	99.69	99.38	-
Alqudah et al. [31]	CNN, SVM, RF	-	95.2	100	-
Lin et al. [32]	CNN(ResNet50)	-	93.1	-	-
Singh et al. [33]	MADE-CNN	93.9	94.4	90.72	-
Sahinbas et al. [34]	VGG, ResNet	80	80	-	-
Zhang et al. [35]	CAAD	-	95.18	70.65	-
Zhou et al. [36]	ResNet-SVM	93.6	93	-	-
S. Nafisah et al. [37]	CNN	99.72	99.82	99.86	-
L. Gaur et al. [12]	Efficient NetB0	88.0	92.93	95	-
**Our proposed**	**ULTRA-X-COVID Net**	**98**	**98**	**98.5**	**96**

## Data Availability

The dataset used for the current study is publicly available at https://github.com/lindawangg/COVID-Net (accessed on 24 October 2023).
